# A place to call home: study protocol for a longitudinal, mixed methods evaluation of two housing first adaptations in Sydney, Australia

**DOI:** 10.1186/s12889-015-1700-y

**Published:** 2015-04-09

**Authors:** Elizabeth Whittaker, Wendy Swift, Paul Flatau, Timothy Dobbins, Olivia Schollar-Root, Lucinda Burns

**Affiliations:** National Drug and Alcohol Research Centre, UNSW, Sydney, New South Wales 2052 Australia; NHMRC Centre of Research Excellence in Mental Health and Substance Use, National Drug and Alcohol Research Centre, UNSW, Sydney, Australia; Centre for Social Impact, Business School, The University of Western Australia, Perth, Australia; Discipline of Pharmacology, Sydney Medical School, University of Sydney, Sydney, Australia

**Keywords:** Homeless, Housing, Health, Drug misuse, Australia, Longitudinal, Mixed methods

## Abstract

**Background:**

This protocol describes a study evaluating two ‘Housing First’ programs, Platform 70 and Common Ground, presently being implemented in the inner-city region of Sydney, Australia. The Housing First approach prioritises housing individuals who are homeless in standard lease agreement tenancies as rapidly as possible to lock in the benefits from long-term accommodation, even where the person may not be seen as ‘housing ready’.

**Methods/Design:**

The longitudinal, mixed methods evaluation utilises both quantitative and qualitative data collected at baseline and 12-month follow-up time points. For the quantitative component, clients of each program were invited to complete client surveys that reported on several factors associated with chronic homelessness and were hypothesised to improve under stable housing, including physical and mental health status and treatment rates, quality of life, substance use patterns, and contact with the health and criminal justice systems. Semi-structured interviews with clients and stakeholders comprised the qualitative component and focused on individual experiences with, and perceptions of, the two programs. In addition, program data on housing stability, rental subsidies and support levels provided to clients by agencies was collected and will be used in conjunction with the client survey data to undertake an economic evaluation of the two programs.

**Discussion:**

This study will systematically evaluate the efficacy of a scatter site model (Platform 70) and a congregated model (Common Ground) of the Housing First approach; an examination that has not yet been made either in Australia or internationally. A clear strength of the study is its timing. It was designed and implemented as the programs in question themselves were introduced. Moreover, the programs were introduced when the Australian Government, with State and Territory support, began a more focused, coordinated response to homelessness and funded rapid expansion of innovative homelessness programs across the country, including Common Ground supportive housing developments.

**Electronic supplementary material:**

The online version of this article (doi:10.1186/s12889-015-1700-y) contains supplementary material, which is available to authorized users.

## Background

Rising rates of homelessness have been well documented across industrialised countries, including the USA [1], the UK [2] and France [3]. In keeping with this trend, recent Australian figures indicated an eight per cent increase in national homelessness since 2006; approximately one in every 200 Australians were homeless in 2011 [4]. Alarmingly, it is estimated that Australian homelessness services provide supported accommodation to only 20 per cent of those who are homeless each night [4].

There is strong evidence to suggest that homelessness is associated with physical and mental health status [5,6]. Individuals experiencing homelessness face higher rates of preventable physical health conditions than the general population, including cardiovascular diseases, infectious diseases and unintentional injuries [6,7]. These conditions are associated with poorer nutrition and living conditions, lack of preventative health measures, limited education, as well as increased levels of psychological stress, exposure to violence and mental health conditions [6,7].

Australian and international evidence highlights the heightened prevalence of substance use disorders and other mental health disorders across homeless populations [6,8-12]. 2007 Australian population data revealed that self-reported rates of a mental health disorder in the past year were notably higher for those respondents with a lifetime homelessness history (54%) compared to those respondents who had never been homeless (19%) [13]. Specifically, substance use disorders were the most common mental health condition amongst the homelessness history respondents, with a reported prevalence rate three times greater than the general population (18% vs. 5%) [13]. Taken together, the heightened comorbidities of physical and mental health conditions experienced by individuals who are homeless places them at elevated risk of involvement with both the health and criminal justice systems [9,10,12,14-18].

Evidence suggests that long-term housing provision improves the health and quality of life of people who are homeless [19,20] and yields substantial cost savings across the health and criminal justice systems [21]. The annual whole-of-government savings are estimated to be at least twice as large as the annual cost of delivering these programs, although the evidence also suggests that the costs of homelessness are uneven across the homeless population [15,22].

### The Australian context

Similar to the international community, homelessness was identified as a key concern by the former Australian Government in 2008. To mark this commitment, the Council of Australian Governments set out a White Paper, *The Road Home* [23], with the headline goals of halving overall homelessness and offering supported accommodation to all rough sleepers by 2020.

Since that time, a range of interventions in the form of assertive outreach services and rapid re-housing programs have been introduced across Australia, including in Sydney, New South Wales (NSW), which has the largest metropolitan homeless population in the country [4]. In response to the geographical concentration of homeless individuals in Sydney, the Inner City Assertive Outreach Program (commonly known as Way2Home) commenced operation in 2010 to provide housing and health assistance to chronic rough sleepers. Jointly managed housing and health support teams used an assertive outreach approach to engage with rough sleepers and coordinate their entry into long-term housing models based on the Housing First approach.

### Housing first

Originally developed in the USA, the philosophy of Housing First is to provide chronically homeless individuals with immediate access to permanent housing without first having to meet traditional pre-requisites such as sobriety or treatment compliance. The provision of housing is seen as the first step and is then combined with coordinated support to enable clients to sustain their tenancies and deal with their often complex needs [24].

### Housing first program 1: scatter site

Platform 70 is a Sydney-based Housing First program that commenced in August 2011. The program is government funded to head-lease 70 units of housing stock from the private rental market scattered across Sydney. Clients housed through Platform 70 were provided wrap-around support services to ensure their tenancies were successfully maintained. Accessing housing from the private rental sector (through a community housing head-lease arrangement) is an innovative approach that increases the housing options available to people sleeping rough. The public housing stock has decreased significantly in Australia and demand for public housing from very low income earners, homeless individuals and their supporting agencies far outstrips supply [25]. The private rental housing market represents a critical, but hitherto under-utilised option for transitioning from homelessness, and the immediacy and diversity of the private rental market can assist with achieving rapid access to housing.

### Housing first program 2: congregated site

The second Housing First program which will be examined in this study is Common Ground Sydney, which opened in November 2011. Common Ground provides social housing accommodation with a community housing provider managing tenancies. This socially integrated housing complex comprises 104 apartments, half of which were allocated to individuals with a chronic homelessness history [23] and the other half to priority social housing and affordable housing tenants. On-site extensive health and social support services are available to all tenants, as well as a link to a social enterprise. The Common Ground model has been rapidly expanded across Australia, with nine other Common Ground buildings either under construction or in operation between 2008 and 2013 [26].

### Aims

While the Housing First approach has been evaluated internationally [27,20,28,29] and is being keenly applied in Australia, there has been a paucity of evidence on the effectiveness of Australian adaptations of the approach. Findings from evaluations of individual Housing First programs within Australia have recently been released publicly [30,31], however no study examining more than one of these adaptations with the same methodology has been conducted. Further, evaluations to date have focused largely on housing sustainability rather that incorporating broader measures of health and quality of life. Given this, there remains a pressing need to determine the efficacy of these respective models (Platform 70 [scatter site]; Common Ground [congregated site]) in relation to both housing and health outcomes. In response to this gap in the evidence-base, we have commenced a longitudinal analysis of these two adaptations using a standardised mixed methods approach to examine the changes in housing and health outcomes associated with the respective models.

Specifically, we aim to:

1. Determine whether changes occurred with respect to clients’ housing stability, physical and mental health and treatment rates, psychological distress, quality of life, substance use patterns, and health and criminal justice system contact.
2. Identify the demographic and social characteristics of clients that predicted success in each model. For the purposes of this study, success was defined as sustained tenancy and improved health and well-being outcomes.
3. Calculate the net of ‘cost offsets’ of the two programs; that is, the reduction in measurable health, criminal justice and other costs borne by government as a consequence of the program.

## Methods/Design

### Study design

The process of allocation to the two housing programs was determined by needs analyses conducted by the case workers who engaged with homeless individuals in the designated catchment area of inner Sydney. Given this process, a control group was not a feasible option and an ecological study was selected which utilised a longitudinal, mixed methods design. Housing and health outcomes were compared at baseline and 12 months follow-up of the scatter site (Platform 70) and congregated site (Common Ground) programs. The methodology, conduct and reporting of this study is in accordance with the Strengthening the Reporting of Observational Studies in Epidemiology (STROBE) initiatives for cohort studies ([32] STROBE checklist can be found in Additional file 1).

This study encompasses three sources of data collected at baseline and 12 months follow-up: 1) client surveys; 2) semi-structured interviews with clients and stakeholders; and 3) housing and support program data.

### Participants, setting and measures

#### Client surveys

Eligibility criteria to participate in the client surveys were that participants had to be at least 18 years old at baseline, were able to provide informed consent, had a history of chronic homelessness (more than six months history of sleeping rough), and were currently housed or waiting for housing with the Platform 70 or Common Ground programs. All individuals housed through these two programs had to firstly be engaged through the Way2Home assertive outreach program, and as such would have received support prior to entering either housing program.

Initially, all eligible clients were asked by their case manager whether they would consent to being approached by a trained researcher to explain the details of the study. All clients who agreed to participate were provided with a Participant Information Statement and Consent Form which they were asked to read and sign. If a participant was unable to read, all information and instructions were read aloud. The survey was then administered by a trained researcher who read the questions to the participant and recorded their responses in a pen and paper format. Upon completion, the survey was sealed in an envelope and returned to the research supervisor for a quality control check (see Additional file 2 for further information on recruitment process).

Surveys were conducted in a neutral public space that was convenient and comfortable for the participant, such as a park, café or an available consultation room at Common Ground. As compensation for their time and travel, participants were offered an AUD$40 food voucher [33]. All researchers completed a three-hour training session on administering the client survey and adhering to an interviewer safety protocol [34]. On average, surveys took 45 minutes to complete.

To reduce loss at follow-up, a systematic process for locating and communicating with participants for the follow-up survey was implemented (see Additional file 3). With their consent, at baseline participants filled out a Locator Form asking them for their primary contact information (e.g., phone number, mailing address, email address) and the contact information for family, friends or service providers with whom they have regular contact. The form provided space for participants to note down any specific instructions for contacting them or their nominated contacts. Participants were also asked to sign a Release of Information Form, which provided consent for the researchers to obtain their contact details from Centrelink (a government agency responsible for welfare payments) if other methods of follow-up were not successful. This form provided space for participants to record their Centrelink client identification number to facilitate record access and matching. Signing the Locator and Release of Information forms were voluntary and a decision not to sign either form did not exclude participants from being involved in the research.

In total, 80 participants were recruited at baseline, which equated to a recruitment rate of 66% (Platform 70: 45 of 70 clients recruited, 64%; Common Ground: 35 of 52 clients recruited, 67%). Reasons for non-recruitment included refusal, unable to make contact, non-English speaking, incarcerated and away during the recruitment timeframe. Baseline data collection occurred from November 2012 to May 2013. As shown in Table 1, the demographic compositions of the two samples are mostly similar to that of all clients within the programs.

**Table 1:** **Demographic composition of the client survey samples recruited at baseline compared to the overall populations**

**Demographics**	**Platform 70 (scatter site)**		**Common Ground (congregated site)**	
	**Sample (n = 45)**	**Population (N = 70)**	**Sample (n = 35)**	**Population (N = 52)**
Sex (% male)	84	83	77	77
Mean age (years)	44	43	44	44
% Indigenous	20	28	10	14
% born in Australia	82	86	73	74

For the 12-month follow-up survey, the participant retention rate was 79% (Platform 70: 37 of 45 participants, 82%; Common Ground: 26 of 35 participants, 74%), which is comparable with longitudinal studies of Housing First programs internationally [29,35]. Follow-up surveys were conducted from October 2013 to May 2014. Nine baseline Platform 70 participants did not complete the follow-up survey due to not being contactable (n = 6), refused (n = 2) or incarcerated (n = 1). Similarly, nine baseline Common Ground participants did not complete the follow-up survey due to not being contactable (n = 6), refused (n = 1), incoherent (n = 1) or passed away (n = 1).

The following measures were collected at both baseline and 12-month follow-up surveys, with the exception of some demographic variables that would not change over time (e.g., sex). To maximise the sample sizes, all eligible Platform 70 and Common Ground clients were invited to participate in the client survey, and recruitment continued until all eligible clients had either provided consent or a reason for non-participation in the research had been ascertained.

### Demographics

Demographic data included sex (male/female), age (years), Indigenous status (yes/no), completed Grade 10 (yes/no), in a relationship (yes/no), number of children, currently employed (yes/no) and cognitive impairment (yes/no). Cognitive impairment was measured using the Montreal Cognitive Assessment (MoCA), which is a standardised screening tool that has been shown to be appropriate for individuals with varying cultural backgrounds, ages and educational levels [36]. Scores range from 0 to 30, with a score of 25 or less indicating a cognitive impairment.

Homelessness and housing history data measures included whether participants had ever experienced the following states of homelessness (yes/no), the total time in each state (12 months or less/more than 12 months), and the age that they first experienced each homelessness state (years): slept rough; emergency accommodation (up to three months); medium to long term accommodation (12 to 24 months); couch surfed; boarding houses or hostels; or in a caravan (other than a holiday). Participants were also asked to quantify how many different periods they had slept rough for six months or more. Lastly, at both baseline and follow-up client surveys, interviewers assisted participants to fill out a housing calendar for the preceding 12 months, which included the type of accommodation and duration of each stay.

### Health

To measure general health, participants reported whether they had ever been diagnosed with the following physical or mental health conditions (yes/no): asthma; emphysema, bronchitis or other respiratory disease; epilepsy or seizure; diabetes; cancer; high blood pressure; stroke; any other heart or circulatory condition; blood-borne virus; liver disease; cellulitis; skin condition; arthritis or joint/muscle disease; eye/vision problems that cannot be corrected by glasses; dental problems; chronic pain; mood disorder; anxiety disorder; psychotic disorder; substance use disorder; personality disorder; eating disorder; or impulse-control disorder. When a health condition was reported, participants were asked if they had received treatment for that condition in the past 12 months (yes/no).

Psychological symptom patterns were measured using the Brief System Inventory (BSI; [37]), a 53-item self-report inventory in which participants rated the extent of distress caused in the past seven days by various symptoms on a five-point Likert scale (‘not at all’, ‘ a little bit’, ‘moderately’, ‘quite a bit’, ‘extremely’). Nine primary symptom dimensions were measured (somatisation, obsessive-compulsive, interpersonal sensitivity, depression, anxiety, hostility, phobic anxiety, paranoid ideation and psychoticism) to provide information on individuals’ psychological status and symptomatology. An overall index of mental health functioning was also generated (Global Severity Index; [37]). The BSI has been used as a valid measure of psychological symptomatology with homeless and vulnerable populations [17,38] and is sensitive to identifying change over time [39,40].

### Quality of life

The World Health Organization’s Quality of Life-BREF (WHOQOL-BREF) instrument measured quality of life across the four domains of physical health, psychological health, social relationships and environment. This standardised 26-item questionnaire asked participants to rate the degree to which they experienced the items in the past two weeks on a five-point Likert scale (low score of ‘1’ to high score of ‘5’), with a higher score indicating better quality of life. The WHOQOL-BREF has been validated as a reliable brief instrument to assess quality of life in homeless individuals across all four domains [41].

### Substance use

Substance use patterns were measured by a number of variables. Lifetime and three-month use of the following substances was recorded (yes/no): tobacco; alcohol; cannabis; amphetamines; cocaine; inhalants; sedatives; hallucinogens; and opioids. Those participants who reported use in the past three months were asked to indicate their frequency of use (once weekly or less/more than weekly), the frequency they had a strong desire or urge to use each substance (once weekly or less/more than weekly), and the frequency that health, social, legal or financial problems resulted from the use of each substance (once monthly or less/more than monthly). Non-medical use of a drug by injection in lifetime (yes/no) and the past three months (yes/no) was recorded. If participants had injected in the past three months, they were asked to rate their frequency of injecting (once weekly or less/more than weekly).

### Service utilisation

To measure health service utilisation, participants were asked if they had attended the following services over the past 12 months (yes/no): inpatient hospital services for physical health; inpatient hospital services for mental health; emergency department (ED) for physical health; ED for mental health; ambulance call-out; drug and alcohol treatment facility; general practitioner (GP) for physical health; GP for mental health; or a specialist mental health service. From this, the total number of different health services utilised over the past 12 months was computed (range 0–8). Participants were asked to report the number of days they had utilised each health service over the past 12 months (range 0–365 days). For example, ‘*In the past 12 months, how many days in total did you stay in hospital because of a physical health problem*?’

Finally, to measure criminal justice system contact, participants reported whether they had been in contact with the following channels of the justice system over the past 12 months (yes/no): held in an adult prison; held overnight by police; attended court for a criminal matter; under supervision of a Parole or other Justice Officer; or stopped by police on the street. From this, the total number of different criminal justice system channels participants had been in contact with over the past 12 months was computed (range 0–5). Participants also reported the number of days over the past 12 months they had contact with each criminal justice system channel (range 0–365 days).

#### Semi-structured interviews with clients and stakeholders

The qualitative component comprised semi-structured interviews to explore the key elements and processes of the service delivery of each model. The design of the client interviews was intended to reflect the different housing arrangements; one-on-one interviews were conducted with Platform 70 clients as they lived independently, whereas group interviews were conducted with Common Ground clients as they resided in a congregated setting. For Platform 70 clients, case managers initially recommended clients who varied in tenancy outcomes (sustained or failed tenancies) to take part in the interviews. All recommended clients were contacted either via the phone or by letter and invited to participate in a qualitative interview about Platform 70. In total, eight clients completed interviews during the evaluation period. Recruitment for the Common Ground client interviews was facilitated by staff who approached formerly homeless residents and informed them of the details of the group interviews. In total, 19 clients participated in a group interview. Participants received an AUD$40 food voucher as reimbursement for their travel and time.

The qualitative interviews with clients comprised a number of open-ended questions to explore their experiences and perceptions of their housing and support services. Topics covered included the circumstances that led to homelessness, the importance of having a home, perceptions of and experiences with housing providers, services and support offered and received, relationships with support services, changes in life since being housed, difficulties encountered, and future goals and aspirations.

A purposive sampling method was used for the stakeholder interviews for both housing programs, which involved targeting stakeholders with experience and knowledge regarding the implementation and operation of the programs. Stakeholder interviews were conducted with community housing providers, front-line and managerial support service representatives, and government and non-government agency staff who were equipped to provide feedback. In total, eight Platform 70 stakeholders and 30 Common Ground stakeholders completed interviews during the evaluation period. Some stakeholders were interviewed at both baseline and follow-up due to their close involvement in the programs and ability to comment on change during the evaluation timeframe (Platform 70: n = 1; Common Ground: n = 12).

Qualitative interviews with stakeholders addressed the facilitators and barriers related to achieving program objectives. Open-ended questions explored the implementation and outcomes of the housing and support, as well as the effectiveness of contractual and partnership structures embedded in the programs. Stakeholders were asked questions relevant to their experience about the process of providing housing, the development and maintenance of support, tenancy management, clients’ needs and independence, and measures of progress and success in the programs.

Clients and stakeholders who agreed to participate in the semi-structured interviews were asked to read and sign a Participant Information Statement and Consent Form. This form requested consent for the research team to audio-record and transcribe the interview for analysis. To protect participants’ privacy, neither the personal identity nor organisational affiliations will be identified in any publications.

#### Housing and support program data

Program data was accessed for the cost analysis. Of all consenting participants, a breakdown per month of their rental arrears, the arrears escalation pathway undertaken by staff, and tenancy damages were extracted. De-identified program data of all clients was also obtained, which included the duration and intensity of support services received (housing case management and health services) and tenancy stability indicators such as the total number of days housed, frequency of housing transitions since first being housed by the program, and number of neighbour complaints during the evaluation timeframe. Agencies providing support and tenancy management to clients as well as community housing providers were interviewed to ascertain the costs of program provision.

### Data analysis

Given the nature of the housing programs under evaluation, we were limited by the number of clients engaged with each program and therefore did not perform a formal power-based sample size calculation. Instead, we invited all eligible Platform 70 and Common Ground clients to participate in the client survey, and recruitment continued until all eligible clients had either provided consent or declined participation in the research.

Loss to follow-up comparisons that assesses the characteristics of participants in the two programs who completed both baseline and follow-up client surveys (completers) against those who only completed the baseline survey (non-completers) will be made. The purpose of these comparisons is to determine whether there are any key differences between survey completers and non-completers of each program. Other patterns of missing data will be examined to determine if there are potential biases. Appropriate literature that addresses how to deal with missing data and small populations and/or small samples will also be consulted.

To address Aims 1 and 2, a profile of clients engaged with the two housing programs will be constructed, which will include demographics, homelessness history and health status. Multivariate analysis will be undertaken to determine changes over time (baseline to follow-up) in key outcomes, including housing, health status and treatment rates, quality of life, substance use patterns, and contact with the health and criminal justice systems within and across the two programs. Additionally, multivariable logistic regression will be used to identify factors associated with sustained tenancies using SAS version 9.4 software.

Qualitative interviews with clients and stakeholders will be thematically analysed in line with the general inductive approach using NVivo 10 software. The analysis will identify key themes in sustained and failed tenancies. These data will be presented in such a way as to give some indication of the commonality or otherwise of particular views, but will not purport to be representative.

To address Aim 3, the cost to government of mainstream health and criminal justice service use by Platform 70 and Common Ground clients and how this use changed over time will be examined. It will also review the cost of delivering the programs and the extent to which this is offset by a decrease in the cost of mainstream government services use by participants.

A detailed examination of mainstream services will utilise the health and criminal justice system contact data collected in the client surveys. Associated costs incurred in the 12 months prior to the baseline client survey will be compared with the cost of services used in the 12 months prior to the follow-up client survey. The cost of health and justice services will also be compared with the NSW population average.

The cost of health and justice services used by client survey participants will be estimated by applying the unit cost for each service to reported use. Unit costs will be estimated from publicly available information including the Report on Government Services 2013 [42] and various other reports, including those published by the Australian Institute of Health and Welfare [43-47]. These sources will also be used to estimate average service use and associated cost for the NSW population.

The cost of delivering the Platform 70 and Common Ground programs will be examined with reference to recurrent expenditure for the period in which clients were housed in the program. We shall also consider the range of rental subsidies provided to clients in the two housing models. Funding and expenditure data will be sought directly from the providers of social, health and tenancy management, and administrative services. To estimate the recurrent cost incurred by housing providers to manage and maintain properties, we shall draw on estimates obtained from the Report on Government Services [42]. Platform 70 and Common Ground case workers potentially provide more time to resolving issues associated with their tenancies than is normal. This implies that the average recurrent cost/dwelling is likely to represent a conservative estimate of the cost incurred by housing providers.

Housing providers also incur the capital cost of accommodation. In our cost analysis we will only consider public and community housing capital costs in the Common Ground program as Platform 70 involves the head leasing of privately owned dwellings. The average capital value of public housing and community housing properties available to Common Ground clients will be established and the annualised value of capital employed estimated by applying an opportunity cost of capital of 8% [42] to the average capital value.

### Ethics, funding and dissemination

Ethical approval was obtained from the NSW Population & Health Services Research Ethics Committee and the UNSW Human Research Ethics Committee. Funding for this evaluation was competitively gained through a tender process from Department of Family and Community Services – Housing NSW. The funding body had no further role in study design; in the collection, analysis and interpretation of data; in the writing of the findings; or in the decision to publish the findings. The findings will be disseminated through government reports, peer-reviewed journals and national and international conference presentations. In future publications we will follow the STROBE guidelines for cohort studies [32].

## Discussion

### Contributions

Given the chronicity of homelessness in Australia, a novel housing policy approach to reducing homelessness has been recently introduced. The housing programs are based on the Housing First approach, whereby individuals who are chronically homeless are offered a tenancy, without first having to be housing-ready. This study will address an important gap in both the public health and housing literature by examining the nature and extent of changes for clients upon being placed in one of the two variations of the Housing First approach; Platform 70 (scatter site model) or Common Ground (congregated model). Additionally, this study will document the journeys of a sample of people in the programs by recording their individual changes over time as they are placed into one of the long-term housing programs available.

Housing and health are inextricably linked and evaluations of housing policy should measure outcomes across both domains. Whilst randomised controlled trials are the gold standard approach for much health research, they are not the most appropriate methodology when assessing the impact of programs that deliver housing and the denial of the intervention (housing) to a control group is not an option. In this situation, a longitudinal mixed-methods approach using standardised and validated measures is appropriate.

### Strengths and limitations

A key strength of this study is that it is uniquely positioned to provide robust evaluative evidence of the efficacy of these housing programs within an Australian context. The timing of this study is particularly critical due to the recent rapid development of Common Ground buildings throughout Australia, despite the lack of published empirical research to support the expansion of the model [26]. A further strength of the methodology is the extensive recruitment and follow-up procedures in place. The success of these procedures to recruit and follow-up with transient clients will provide a template for future research with vulnerable and homeless populations.

It is important to acknowledge the limitations of this evaluation. First, individuals housed through these programs had chronic homeless histories and were identified as requiring priority housing due to their high level of vulnerability. Subsequently, the findings have limited generalizability to the full homeless population in Sydney. Second, the methodology is mostly reliant on self-report measures, which may affect reporting accuracy due to factors such as memory error, nondisclosure, social desirability or intentional misrepresentation [48]. To reduce the chance of this occurring, a recall timeframe of 12 months or less and visual show cards were used, as were standardised tools validated for use with vulnerable populations [49]. It was noted during the study design that previous studies have demonstrated a moderate to high internal consistency in self-reports by individuals experiencing homelessness, including those with severe mental health conditions [50,51]. Additionally, all participants were informed that their client surveys and qualitative interviews were confidential and their responses would not affect their relationships with housing or support services. The final limitation is the small sample sizes. However, the nature of the programs means that the population from which to recruit from was also small. Despite this, substantial time and resources were devoted to contacting every eligible client to ensure that everyone had an opportunity to participate, including going on street patrols and spending days shadowing case managers. Significant efforts have also been made to identify appropriate analytical techniques for small samples.

## Conclusion

This study is the first to utilise a standardised mixed methods approach to simultaneously evaluate two adaptations of the Housing First approach in Australia. This extends previous work that has focussed largely on housing sustainability as the outcome. This research has the capability to contribute substantially to both public health and housing policy realms, and is significant in its timing given the rising rates of homelessness and expansions of similar housing programs in Australia and worldwide.

## Additional files

Additional file 1:
**STROBE Statement for reports of cohort studies.**
Additional file 2:
**Flowchart of recruitment process for client survey participants.**
Additional file 3:
**Flowchart of follow-up process for client survey participants.**
