# Supplementary figures and images for: A place to call home: study protocol for a longitudinal, mixed methods evaluation of two housing first adaptations in Sydney, Australia
Source: BMC Public Health. 2015 Apr 9;15:342. doi: 10.1186/s12889-015-1700-y (PMC4393870; doi:10.1186/s12889-015-1700-y)

1 **Additional File 2: Flowchart of recruitment process for client survey participants**

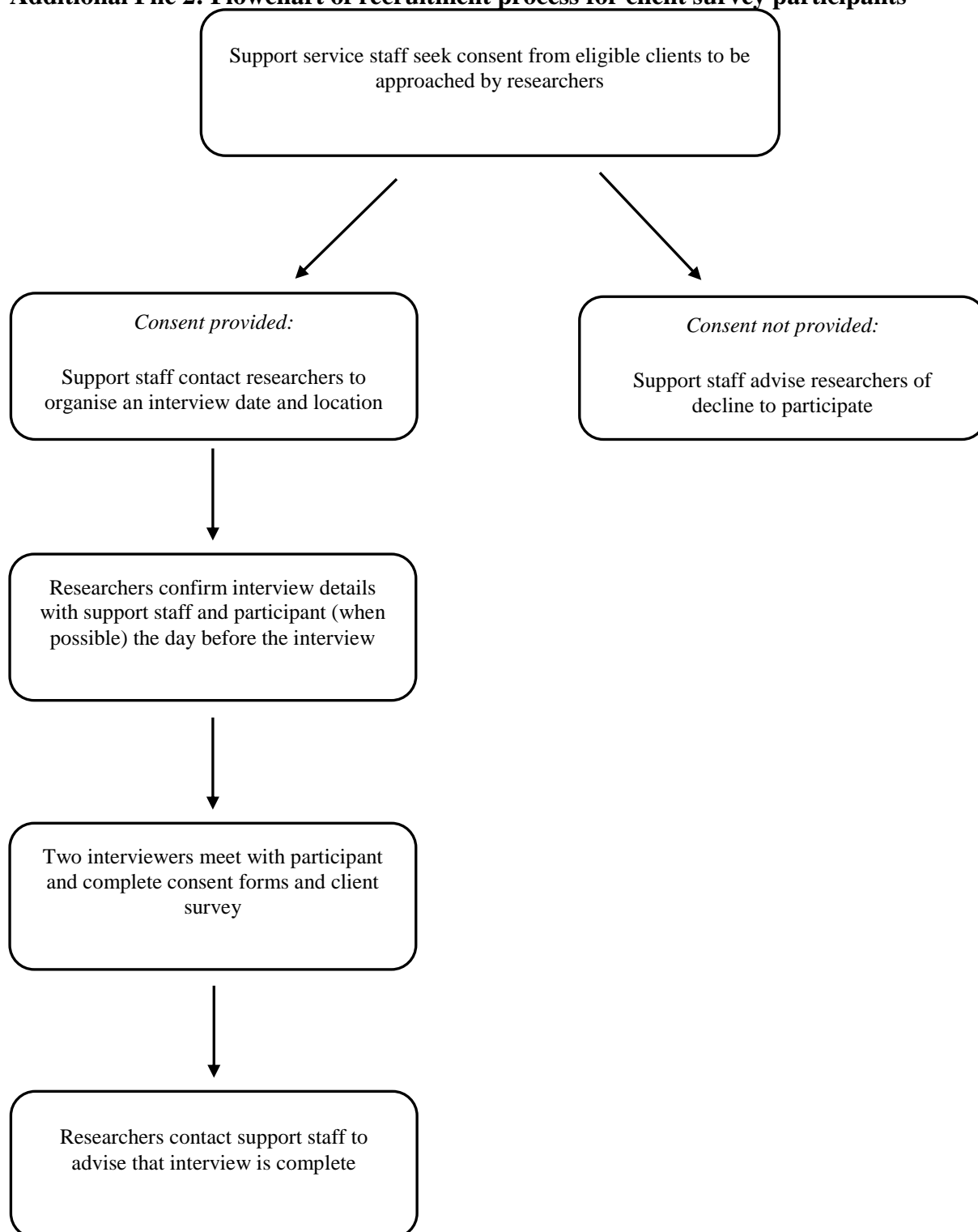

2  
3  
4  
5  
6  
7

Supplement: Additional file 2: — Flowchart of recruitment process for client survey participants. [file 12889_2015_1700_MOESM2_ESM.pdf]

1 **Additional File 3: Flowchart of follow-up process for client survey participants**

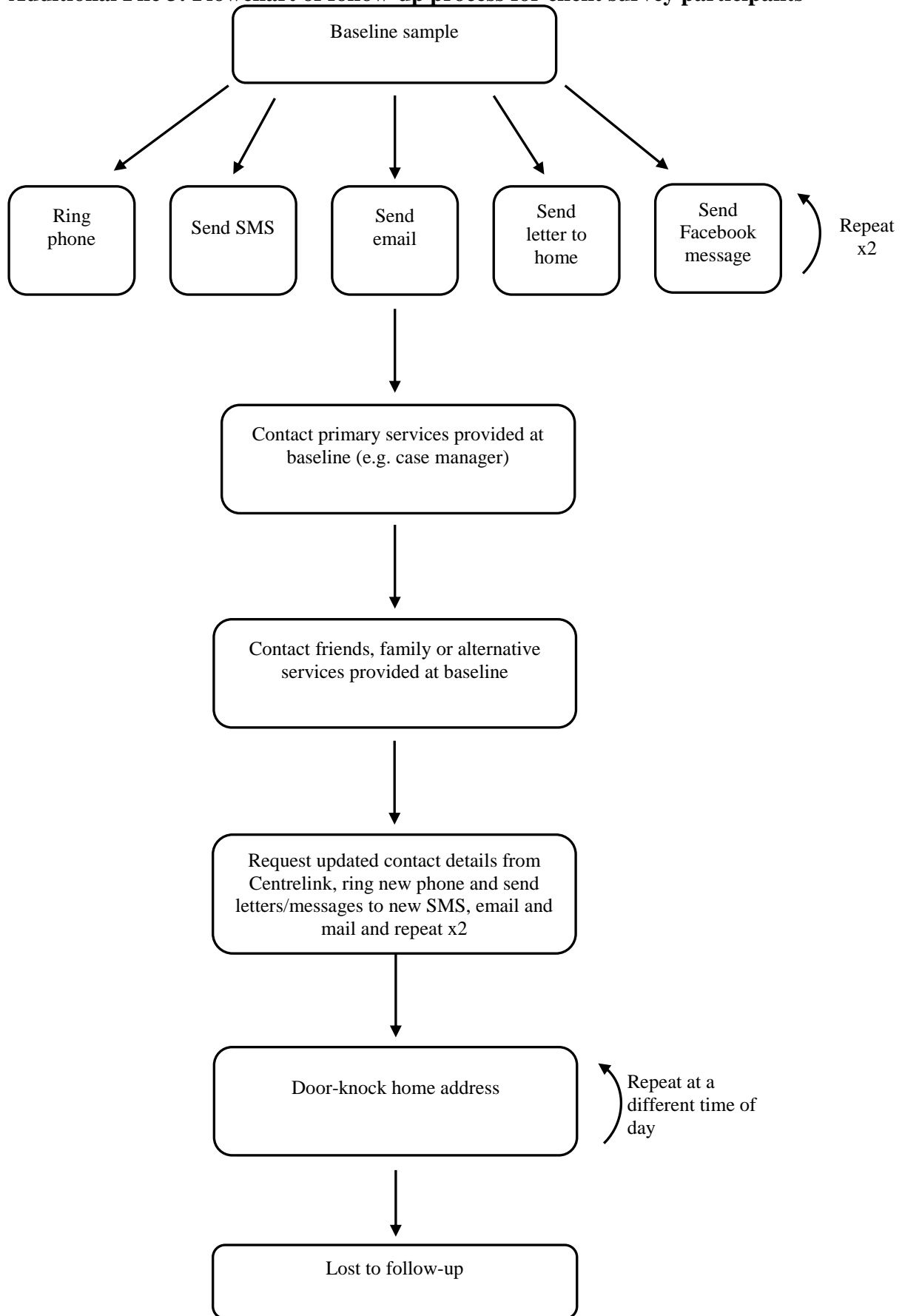

Supplement: Additional file 3: — Flowchart of follow-up process for client survey participants. [file 12889_2015_1700_MOESM3_ESM.pdf]
